# Exploring the structural characteristics of the Adult Social Care Outcomes Toolkit (ASCOT) and ASCOT-Carer

**DOI:** 10.3310/nihropenres.13259.2

**Published:** 2023-03-21

**Authors:** Stacey Rand, Ann-Marie Towers, Juliette Malley, Barbora Silarova

**Affiliations:** 1 Personal Social Services Research Unit, University of Kent, Canterbury, UK; 2 Centre for Health Services Studies, University of Kent, Canterbury, UK; 3 Care Policy and Evaluation Centre, London School of Economics and Political Science, London, UK

**Keywords:** quality of life, social care, long-term care, ASCOT, service users, carers

## Abstract

**Background:**

Measurement models inform the approach to assess a measure’s validity and also how a measure is understood, applied and interpreted. With preference-based measures (PBMs), it is generally accepted that they are *formative*; however, if they are applied without preferences, they may be *reflective*, *formative* or *mixed*. In this study, we sought to empirically test whether the *reflective*, *formative* or *mixed* measurement model best describes PBMs of social care-related quality of life (SCRQoL) – specifically, the ASCOT and ASCOT-Carer. We also explored the network approach, as an alternative.

**Methods:**

ASCOT and ASCOT-Carer data were analyzed using confirmatory factor analysis and Multiple Indicators Multiple Causes models to test reflective, formative or mixed measurement models, respectively. Network analysis of partial correlations using the Gaussian graphical model was also conducted.

**Results:**

The results indicated that the reflective measurement model is the worst fit for ASCOT and ASCOT-Carer. The formative or mixed measurement models may apply to ASCOT. The mixed measurement model was the best fit for ASCOT-Carer. The network analysis indicated that the most important or influential items were *Occupation* and *Personal cleanliness and comfort* (ASCOT) and *Time and space* and *Self-care* (ASCOT-Carer).

**Conclusions:**

The ASCOT and ASCOT-Carer are best described as formative/mixed or mixed measurement models, respectively. These findings may guide the approach to the validation of cross-culturally adapted and translated versions. Specifically, we recommend that EFA be applied to establish structural characteristics, especially if the measure will be applied as a PBM *and* as a measure of SCRQoL. Network analysis may also provide further useful insights into structural characteristics.

## Plain English summary

For many people living with long-term health conditions or disabilities, community-based social care services (like, home care) enable them to maintain independence, stay connected, and to live well. For families and friends who care for someone (‘carers’), these services may also help them. They may allow carers to continue in paid employment and to have time for hobbies, friendships, to stay healthy, and connect with others in a similar situation. An important question is what type(s) of community-based services, best support people and their carers.

To find this out, we need a way of measuring the effect services have on people’s lives. The Adult Social Care Outcomes Toolkit (ASCOT) is a questionnaire that asks people about aspects of their life that might be affected by social care services (for example, having control over everyday life). This questionnaire has already been used by researchers and care providers to review how well social care services support people. There is also another version of the questionnaire called the ASCOT-Carer, which looks at aspects of life that are important to carers.

There has been interest in culturally adapting and translating these measures into other languages. However, there are different ways of establishing how well a translated version relates back to the original. This is important to make sure that the new version is measuring what we expect it to.

In this paper, we compare different ways of understanding the information collected using the ASCOT and ASCOT-Carer in England. This will inform how to approach the testing of ASCOT (and other similar measures) that have been translated into new languages. It also helps us to understand how different aspects of life that are supported by social care services are related to each other. This can inform our understanding of people’s needs and how to best support them.

## Introduction

The theoretical and philosophical questions of measurement models are important to psychometric research. Implicitly and explicitly, they inform the approach to assess a measure’s validity and also how a measure is understood, applied and interpreted. In this paper, we will draw on a preference-based measure (PBM) used in economic evaluation of long-term care services, the Adult Social Care Outcomes Toolkit for adults using care services due to long-term health conditions or disability (ASCOT) ^1^ and their unpaid (family) carers (ASCOT-Carer) ^2, 3^, to illustrate the issues related to applying measurement models to PBMs. In doing so, we will highlight key implications for the development (especially, the validation of translated and cross-cultural adapted measures) and application of PBMs in research, evaluation and practice.

**Figure 1.**
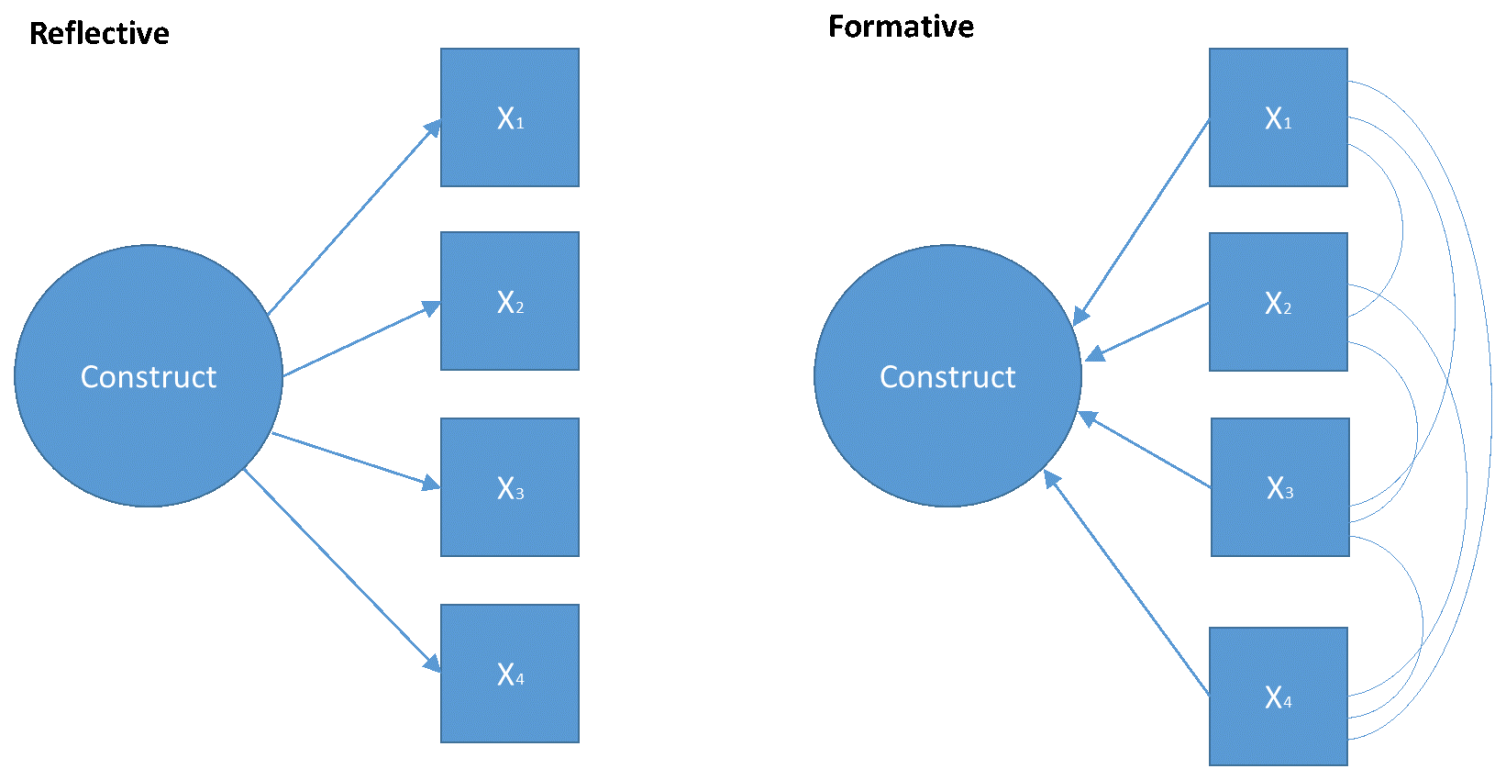
Formative and Reflective Measurement Models.

The two measurement models commonly applied in psychometric research are *reflective measurement models* (RMM) and *formative models* (FM) (see Figure 1). In RMM, the construct is the common cause of items ( *observables*), *i.e.* the relationships between the items are due to a common causal path to the construct, not interrelationships between the items. Compared to RMM, the items in FMs are conceptualized as a set of independent measures that come together to form a construct. The items may be inter-correlated. These models also differ in underlying philosophical assumptions. RMMs are based on a realist stance; the construct is conceptualized as being ‘out there’, but unobserved. By contrast, FMs are based on a constructivist position; the construct is a rational construction of the mind and is a theoretical composite of its constituent parts.

Typically, PBMs used in economic evaluation of health and social care services (also known as long-term care outside of the UK), like the EQ-5D and ASCOT, are understood using the FM due to the composite nature of PBMs ^4, 5^. Each item represents a dimension of health or care-related quality of life (QoL) that is distinct from the other items (the *observables*). The PBM index score represents an individual’s outcome state (the *construct*). It is a composite function of the preference weights assigned to each item. The items for PBMs are usually selected on the basis that they will be weakly associated, to avoid redundancy and also to allow tradeoffs between items. Therefore, the proposed methodology for evaluating PBMs, based on the FM, focuses on content validity, face validity and construct validity of the descriptive system (without preferences), rather than structural validity ^5^. The justification for this approach is that the classical test theory (CTT) method of establishing structural validity using exploratory factor analysis (EFA), which may be applied for initial development of new measures and/or the validation of translated and cross-culturally adapted versions of existing measures, is theoretically based on the RMM ^6, 7^. It has been argued that these methods are irrelevant and potentially misleading for measures based on the FM ^8, 9^, even if other CTT methods ( *e.g.* confirmatory factor analysis) may be applied ^7^.

However, PBMs, like ASCOT, may be used in a range of contexts. The ASCOT and ASCOT-Carer have been used as PBMs in economic evaluation ^10– 12^; however, they have also been used in non-economic contexts, without preference weights, *e.g.* for needs assessment, care planning ^13, 14^. Correspondingly, psychometric testing of these measures have taken a combination of RMM ( *i.e.* using EFA) ^1, 2^ and FM ( *i.e.* not applying structural validity methods) approaches ^15, 16^, typically influenced by the view of how the measure will be applied ( *i.e.* as a PBM, or not). Inconsistent approaches to psychometric evaluation of the same measure have given rise to discussion of the correct approach to the translation and cross-cultural validity or adaptation of the measures for new contexts or populations ^15– 17^. One way of resolving this question is to establish empirically which measurement model, RMM or FM, most accurately describes the ASCOT and ASCOT-Carer.

In recent years, however, there have been advances in thinking about measurement models, beyond RMM and FMs. Instead, some measures may be best described by *mixed measurement models*, with a combination of formative *and* reflective relationships between construct and observables ^18^. It has been proposed that measures based on mixed measurement models may be treated as RMMs ( *i.e*. by applying EFA) in psychometric research ^8^. However, there has been critique of limitations of RMM, FM and mixed measurement models, which broadly apply also to the care-related QoL measures, ASCOT and ASCOT-Carer. For example, RMMs are based on the assumption that the observables are locally independent when controlling for the latent variable. This is unlikely to hold for ASCOT measures, since we expect there to be associations between observables (ASCOT or ASCOT-Carer items), aside from an underlying association with the latent construct (social care-related quality of life: SCRQoL), *e.g*., it is likely that a person’s sense of control over their daily life would be directly affected by whether they feel they are doing things they value and enjoy; and *vice versa*. It is also conceptually implausible to say that the items have a common cause ( *i.e*. that having a poor SCRQoL will *result in* having poor control over daily life, personal safety, *etc.*), especially as ASCOT is a PBM where the items were selected to vary independently. Aside from general critique of FMs and their limitations, especially in application to psychological measurement (for example ^19– 21^), a key limitation of FMs is that relationships between observables in FMs are modelled as noise. Where there is justification for expected relationships between observables, as outlined above for ASCOT, the formative approach potentially overlooks important structural information ^7, 22^.

An alternative to RMMs, FMs or their combination in mixed measurement models, is the *network model* (NM). NMs avoid the limitations of FMs, RMMs and mixed measurement models by proposing instead that the construct is a network of causally-related elements (nodes), without any assumptions about the nature or causal direction of relationships ^7^. NMs do not require the existence of latent variable(s), since the construct is still ‘real’ as a complex network or system of interrelated variables ^23^. The NM is based on a critical realist position, *i.e*. that the construct *is* the observed variables in their complex interrelationships of mutual influence ^24^. NMs have been applied in psychological measurement of intelligence ^25^, personality ^26^ and psychological comorbidity ^27, 28^ and has been used in health psychology research, as they enable modelling of complex interdependencies between factors that may affect an outcome ^29^. NMs have also been proposed as a method for psychometric analysis of health-related QoL measures, to address the limitations of both FM and RMMs when applied to measures of health-related QoL ^7^.

The aim of this study was to establish which measurement model best describes the ASCOT and ASCOT-Carer, respectively: *reflective*, *formative* or *mixed*. While the development and psychometric assessment of the ASCOT instruments has applied either the FM ^16^ or the RMM ^1, 2^, these are directly compared here, alongside also mixed measurement models. This will inform approaches to the future validation and development of ASCOT or related PBMs of care-related QoL, especially in translation or cross-cultural adaptation of PBMs. The NM was also explored, as an alternative to RMMs, FMs and mixed measurement models. The purpose was to establish whether it provides further insight into the structure of ASCOT and ASCOT-Carer, beyond what is offered by factor analysis, as a network of mutual interrelationship between items.

## Methods

### Sampling and data collection

To explore the internal structural characteristics of ASCOT and ASCOT-Carer, we conducted secondary analysis on data from two cross-sectional studies in England. These are described below. **Study One** collected data using the standard self-completion version (SCT4) of ASCOT ^1, 30^. **Study Two** collected data from carers of people with dementia about their own QoL outcomes (ASCOT-Carer SCT4 ^2^). To reflect the specific needs and experiences of carers, the ASCOT-Carer has a different set of attributes to the service user versions, with some overlapping domains (see Box 1).

**Box 1.** SCRQoL attributes

ASCOT	ASCOT-Carer
Control over daily life	Control over daily life
Occupation ( *doing things I * *value and enjoy*)	Occupation ( *doing things I * *value and enjoy*)
Social participation and involvement	Social participation and involvement
Personal safety	Personal safety
Food and drink	
Accommodation comfort and cleanliness	
Personal comfort and cleanliness	
Dignity	
	Self-care ( *being able to look * *after myself*)
	Time and space to be myself
	Feeling supported and encouraged in caring role

**Study One: Identifying the Impact of Adult Social Care (IIASC)**

The Identifying the Impact of Adult Social Care (IIASC) study was an interview survey of 990 adults who used community-based adult social care services (e.g. homecare) due to long-term conditions or disability in England. The survey was conducted in 22 local authorities (LAs) between June 2013 and March 2014. The sample was identified from records held by LAs or home care providers. The inclusion criteria were: aged 18 years or over, living in their own home, and receiving support due to physical disability or sensory impairment or mental health conditions or learning disabilities. Because the questionnaire for people with learning disabilities used an adapted easy read version of ASCOT, these data ( *n*=220) are excluded from the analysis presented here.

Eligible participants were invited to participate in an interview, which was either completed face-to-face (74.2%) or by telephone (25.8% of the sample (n=770)). Written or verbal consent was obtained before all interviews. Data were collected on the respondent’s personal characteristics, social care needs, health, type and intensity of service use, informal support from family/friends, and quality of life outcomes, including the ASCOT ^1^. Further details of the questionnaire content and data collection methods are outlined elsewhere ^30^.

**Study Two: Measuring the Outcomes of People with Dementia and their Carers (MOPED) study**

The MOPED study was an observational cross-sectional study to establish the psychometric properties of the ASCOT-Proxy and ASCOT-Carer. The data were collected using self-administered questionnaire (either postal questionnaire or an online version in Qualtrics) among 313 unpaid family carers in England. The inclusion criteria were carers, who provided unpaid help or support to a relative, partner/spouse or friend living with dementia, who used community-based social care ( *e.g*. home care, day centre), was not in residential or nursing care, and unable to self-complete a structured questionnaire, even with help.

Participants were recruited between January 2020 and April 2021 through *Join Dementia Research* (an online opt-in volunteer panel), local carers’ support organisations, healthcare settings and social media. Written informed consent was obtained from all participants. The questionnaire collected data on the respondent’s characteristics, caregiving situation and care-recipient characteristics. SCRQoL was measured by the ASCOT-Carer.

### Statistical analysis

**Figure 2.**
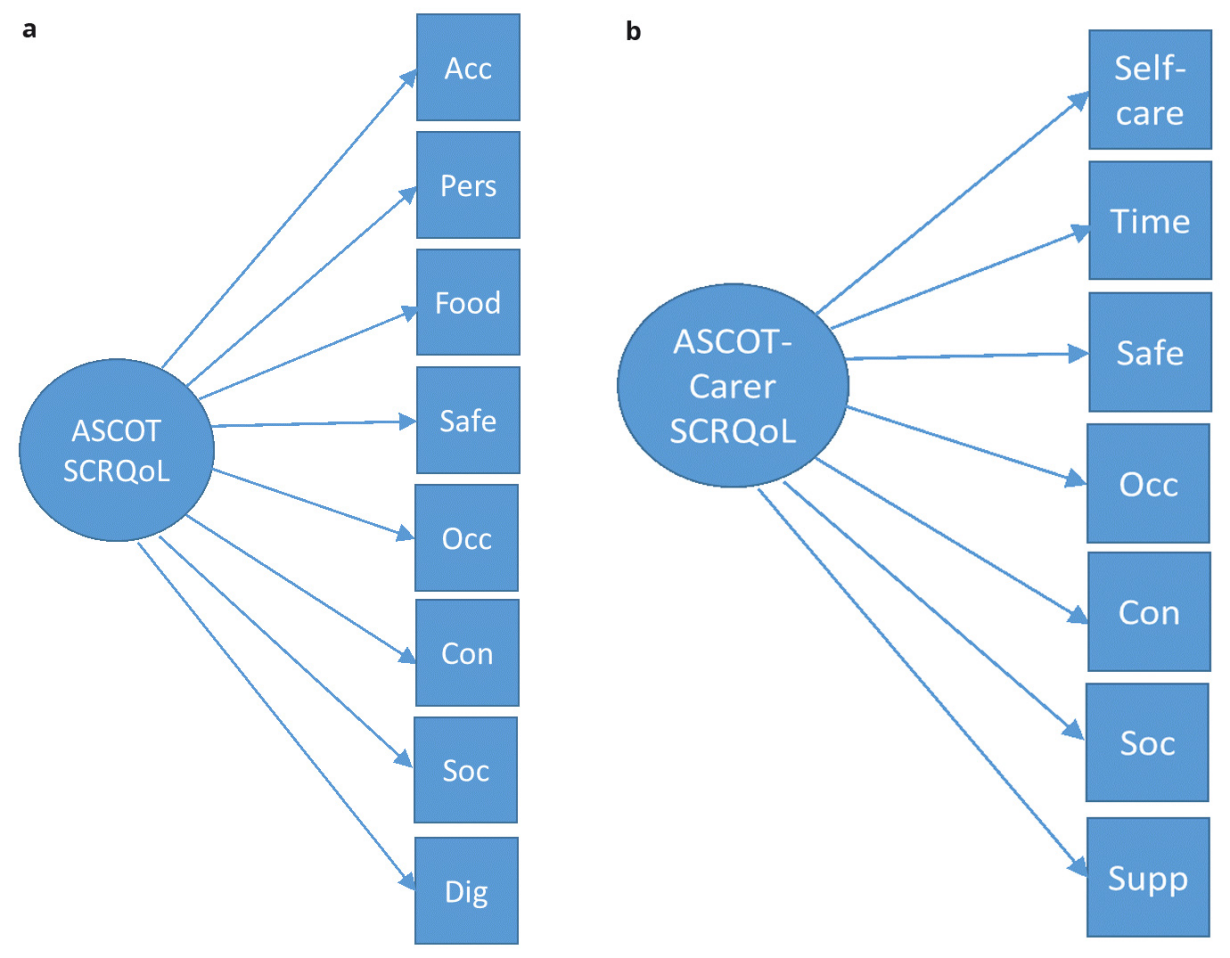
**a. ** Reflective Model for ASCOT. **b.** Reflective Model for ASCOT-Carer.

The first aim of the study was to empirically compare the three measurement models ( *reflective*, *formative* and *mixed*) for ASCOT and ASCOT-Carer, to determine which fits best. The *reflective measurement model* was evaluated using confirmatory factor analysis. Each of the eight ASCOT and seven ASCOT-Carer items were tested separately as reflective indicators of a single latent construct ( *i.e*. SCRQoL -> ASCOT or ASCOT-Carer items – see Figure 2). A single factor was applied based on previous studies of the ASCOT and ASCOT-Carer using exploratory factor analysis (EFA) ( 1; 12) and an exploratory factor analysis of the ASCOT and ASCOT-Carer using the IIASC and MOPED datasets (see Appendix).

**Figure 3.**
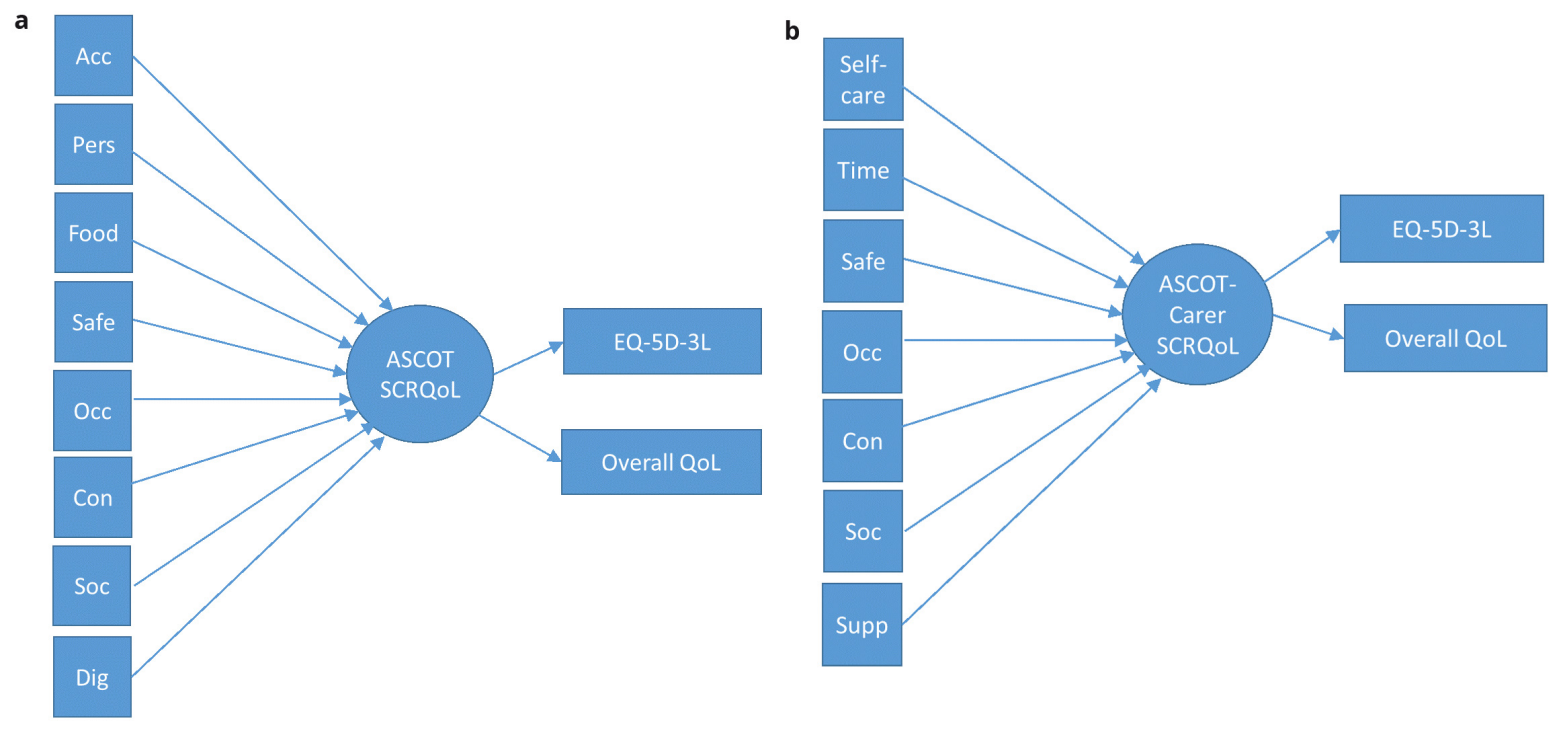
**a**. Formative Model for ASCOT. **b**. Formative Model for ASCOT-Carer.

The *formative* and *mixed measurement models* were evaluated using Multiple Indicators Multiple Causes (MIMIC) models ^31^. These are structural equation models (SEMs) that allow the simultaneous modelling of reflective items that relate to one or more latent variable(s), alongside the relationship between formative items and the latent variable. In the *formative model*, all eight or seven items of the ASCOT or ASCOT-Carer, respectively, were modelled with a formative relationship to the latent variable, SCRQoL. To enable the empirical testing of the models, it is necessary to specify also two or more reflective indicators. These are typically measures of the same or similar constructs. This provides external anchoring against validated measures or items (i.e. EQ-5D, QoL item), when all of the ASCOT items are considered as a composite of SCRQoL (i.e. ASCOT or ASCOT-Carer items -> SCRQoL -> EQ-5D, overall QoL – see Figure 3). Since there are no other validated measures of social care-related QoL than the ASCOT or ASCOT-Carer, measures of the related constructs of health-related QoL and overall QoL were selected. From previous research, these measures are known to be related to ASCOT SCRQoL ^1, 2, 12, 32^. Specifically, for ASCOT, the EQ-5D-3L and a single item 7-point rating of overall QoL were considered. For ASCOT-Carer, the EQ-5D-5L converted by cross-walk to EQ-5D-3L values ^33^ and a single-item 5-point overall rating of QoL were considered. These differences in the measures were due to the available data in each study dataset.

**Figure 4.**
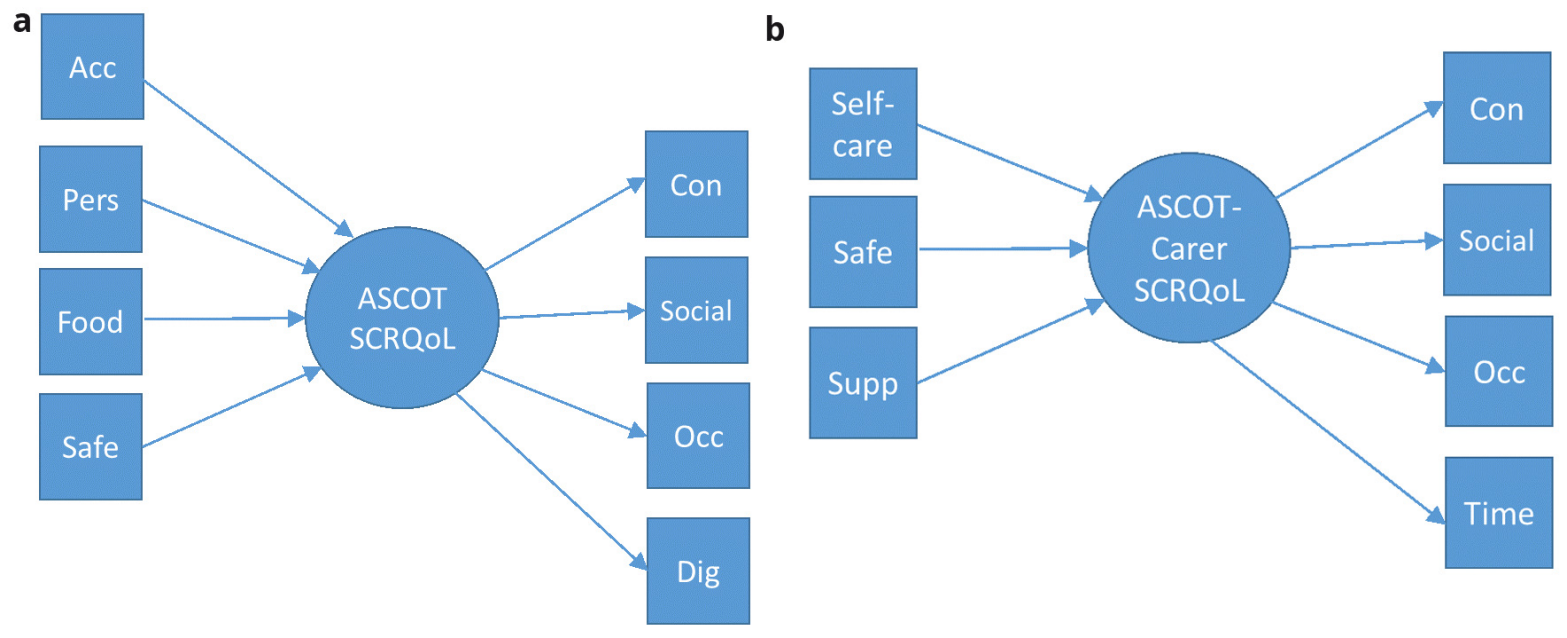
**a.** Mixed measurement model for ASCOT. **b.** Mixed measurement model for ASCOT-Carer. **Key to Figures 2–4:** Acc, accommodation; Pers, personal comfort and cleanliness; Food, food and drink; Safe, personal safety; Occ, occupation; Con, control over daily life; Soc, social participation; Dig, dignity; Self-care, self-care; Time, time and space to be yourself; Supp, feeling supported and encouraged in caring role.

In the *mixed measurement models* (see Figure 4), the ASCOT or ASCOT-Carer items were considered as formative or reflective. These were selected based on theory. In the development of ASCOT, it has been noted that the domains may be categorized as those that relate to: (1) *basic domains* that relate to basic care needs/support to sustain life and health ( *i.e*. Food and drink, Personal or Accommodation comfort and cleanliness, Personal safety (ASCOT); Self-care, Feeling supported in the caring role, Personal safety (ASCOT-Carer)); (2) *higher order domains* that relate to aspects of QoL beyond basic care needs and/or relate to a person’s sense of self and identity ( *i.e*. Control over daily life, Occupation, Social participation (ASCOT and ASCOT-Carer); (3) domains related to how the delivery of care affects a person’s sense of self and identity (Dignity (ASCOT) or Time and space to be yourself (ASCOT-Carer)) ^1^. The *basic domains* were considered as formative, since they may be conceptualized as constituent parts of social care-related QoL ( *i.e*. aspects of QoL that make up the construct, SCRQoL). The *higher order domains* and the *domains related to sense of self and identity* were considered as reflective of SCRQoL ( *i.e*. they are driven by a common factor, SCRQoL).

To compare these models, standardized factor loadings and coefficients were reported for the CFA and MIMIC, respectively, to explore the relationship between items and the latent variable. Model fit statistics were calculated to evaluate the estimated models. The following criteria were applied to indicate good fit: root mean square error of approximation (RMSEA) ≤0.06 (upper 90% confidence interval of ≤0.08), standardized root mean square residual (SMSR) of ≤0.08, with a comparative fit index (CFI) and Tucker-Lewis index (TLI) of ≥0.95 ^34^. In addition to the raw goodness-of-fit statistics, we also report the bootstrap estimation of the 95% (bias corrected) confidence intervals. These were calculated using the *bootstrap* command with 1,000 repetitions.

In addition to comparing and evaluating RMM, FM and mixed measurement models, we also applied network analysis to determine whether this approach, which has been proposed as a suitable approach for measures of health-related QoL ^7^, offers additional useful insights when applied to social care-related QoL measures, like ASCOT. In network analysis, the focus is on the variables ( **nodes**) and the relationships between them ( **edges**). These may be **directed**, which indicate a one-way effect, or **undirected**, which indicates an unspecified mutual relationship.

In this analysis, the ASCOT and ASCOT-Carer items were considered as the **nodes** and all **edges** were specified as undirected. The network was estimated by analyzing partial correlations using the Gaussian graphical model. Polychoric correlation coefficients were applied since the data were ordinal. The coefficients are estimates of the strength of relationship between variables (the ASCOT or ASCOT-Carer items) whilst controlling for the effects of other measured variables in the model. A graphical least absolute shrinkage and selection operator ( *glasso*) approach was applied to the estimation of the correlation network ^35^. This statistical technique takes into account the model complexity and seeks to reduce the number of spurious relationships by reducing small weak edge estimates to zero. The *glasso* tuning parameter (ʎ) may be set from 0 to 1. Increasing the tuning parameter will minimize spurious edges, however, relevant edges may also be suppressed ^29^. The analysis applied ʎ=0.25.

After the models were estimated, the network properties were evaluated. The centrality of nodes ( *i.e*. their relative importance) in determining the network structure was assessed by the number of connections incident to the node ( *degree centrality*). Centrality indices were also calculated and reported for node *strength*, *closeness* and *betweenness* ^29^. The strength index is a composite measure of both the number and strength of connections to a node. The closeness index represents the relationship between one node and the other nodes through its indirect connections ( *i.e*. its connectedness or connectivity). A high closeness index indicates that the node is affected quickly by changes to the other nodes in the network. The betweenness index indicates the importance of a node in relation to the average pathway between other nodes ^29^.

The descriptive statistics, CFA and MIMIC were calculated in STATA version 16. The network analysis was conducted in R.

## Results

The sample characteristics are outlined in Table 1. The sample for study 1 (IIASC) was users of community-based social care services (e.g., home care) due to long-term health conditions or disability. Just over half the sample (52.7%) were aged 65 years or older and 42% were male. There was a wide range of social care needs in the sample, with 9.4% of respondents reporting no needs for activities of daily living. (The study sample included users of services for support with mental health difficulties, where the eight activities of daily living (ADLs) may not relate to the person’s needs.) The overall health-related quality of life of the sample was relatively low (mean EQ-5D-3L index of 0.27), which reflects the study inclusion criteria of using community-based adult social care services.

**Table 1.** Sample characteristics.

	Study 1 (n=770)	Study 2 (n= 313)
	N (%)	N (%)
Male	323 (42.0%)	76 (24.3%)
Age ≥65 years	406 (52.7%)	137 (43.8%)
Ethnicity: white British	704 (91.4%)	296 (94.6%)
Self-reported health: *good or very good*	228 (29.6%)	229 (73.2%)
*fair*	311 (40.4%)	72 (23.0%)
*bad or very bad*	230 (29.9%)	11 (3.5%)
*missing data*	1 (0.1%)	1 (0.3%)
Care recipient’s ADLs with difficulty ¹:		
*none*	72 (9.4%)	0 (0%)
*1–4*	293 (38.1%)	123 (39.3%)
*5–7*	243 (31.6%)	86 (27.5%)
*All 8*	159 (20.6%)	101 (32.2%)
*missing data*	3 (0.4%)	3 (1.0%)
Hours of inf care / week: 0–19 hours	n/a	95 (30.4%)
20–34 hours	n/a	36 (11.5%)
35–50 hours	n/a	32 (10.1%)
≥50 hours	n/a	147 (47.0%)
*missing data*	n/a	3 (1.0%)
	**Mean** **(Std. Dev., Range)**	**Mean** **(Std. Dev., Range)**
Overall QoL	4.43 (1.26, 1 to 7)	3.46 (1.04, 1 to 5)
EQ-5D-3L Index	0.27 (0.39, −0.594 to 1)	0.79 (1.04, −0.594 to 1)

¹ This is a count of activities of daily living (ADLs) where the respondent (or care recipient by proxy report) had difficulty or was unable to complete the task alone, without help.

The study 2 (MOPED) sample were all carers of someone with dementia. The majority of the MOPED sample (study 2) were caring for a parent (n=152, 48.6%) or a spouse or partner (n=130, 41.5%). Most carers were co-resident with the person they support (n=181, 57.8%). The high level of social care needs of care recipients is reflected in the profile of difficulty with ADLs; 32.2% of the sample reported that the person they supported had difficulty with all eight ADLs. Almost half of the sample (47.0%) were carers providing 50 or more hours of unpaid care per week.

**Figure 5.**
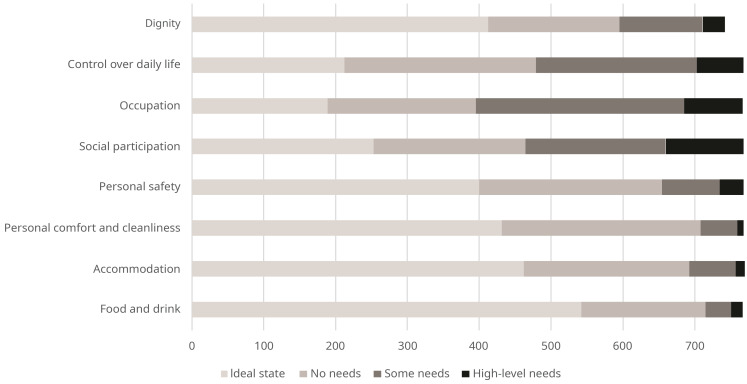
ASCOT ratings (Study 1).

**Figure 6.**
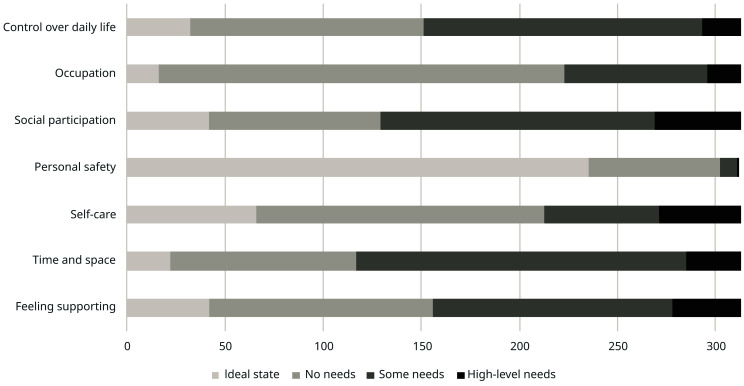
ASCOT-Carer ratings (Study 2).

The distribution of scores by item for ASCOT (IIASC, Study 1) and ASCOT-Carer (MOPED, Study 2) are shown in Figure 5 and Figure 6. The rating of high-level or some needs are highest for the three ASCOT ‘higher order’ domains of Control over daily life, Social participation and Occupation. The ideal state (best care-related QoL) was rated by over half of the sample for the basic domains of Personal comfort and cleanliness (56.1%), Accommodation (60.1%) and Food and drink (70.1%).

The ASCOT-Carer ratings had a different response profile. With the exception of Personal safety (3.2%), between 28% (Occupation) and 63% (Time and space) of ratings per attribute were high-level or some needs. This is broadly consistent with a 2013/14 data collection from carers in England using the ASCOT-Carer ^2^. However, the current study sample had a higher profile of need with regard to Control over daily life (51.8% *versus* 37.0%) and Social participation (58.8% *versus* 33.3%). The reverse was the case for Occupation (28.8% *versus* 49.1% of the sample reported high-level or some needs) ^2^. This is unsurprising given that the study sample was carers of people with dementia living in their own homes. This subgroup of carers are known to have specific high-level social care support needs that relate to the condition, for example, in its fluctuating and unpredictable nature ^36– 38^. The data collection also coincided with the COVID-19 pandemic and associated restrictions in England, which may have affected a number of aspects of quality of life due to the legal restrictions. The lower levels of QoL for Social participation and Control over daily life may, at least partly, be affected by the legal restrictions on socializing, travel/movement, leaving the home and other aspects of life designed to curb the spread of the infection.

The results of the reflective, formative and mixed measurement models are shown in Table 2. The reflective models were a poor fit against the criteria ( *i.e*. RMSEA ≤0.06 (upper confidence interval ≤0.08), SMSR ≤0.08, and CFI/TLI ≥0.95 ^34^). Only SMSR ≤0.08 was met for ASCOT; SMSR ≤0.08 and CFI/TLI ≥0.95 were met for ASCOT-Carer. All of the applied criteria were met for the ASCOT formative model. The chi-square (χ²) test of predicted against observed data was also not significant, which indicates good model fit. By contrast, the formative model was a poor fit for ASCOT-Carer. Only SMSR ≤0.08 was met. Finally, the mixed measurement model was a good fit for ASCOT and ASCOT-Carer. Taken together, the findings indicate that the RMM is the worst fit for ASCOT. The FM or mixed measurement models may apply to ASCOT, with the best fit for the FM. The mixed measurement model was the best fit for ASCOT-Carer.

**Table 2.** Confirmatory factor analysis ( *reflective model*) and MIMIC ( *formative and mixed measurement models*).

	Confirmatory factor analysis (reflective)				MIMIC (formative)				MIMIC (mixed)			
	Study 1: ASCOT		Study 2: ASCOT- Carer		Study 1: ASCOT		Study 2: ASCOT- Carer		Study 1: ASCOT ^1^		Study 2: ASCOT- Carer ^2^	
Measure Domain (if applicable) *Basic, higher or identity* *& sense of self (if* *applicable)*	Factor loading	SE	Factor loading	SE	Stand. Coeff.	SE	Stand. Coeff.	SE	Stand. Coeff.	SE	Stand. Coeff.	SE
**ASCOT** Accommodation *Basic*	0.516 **	0.034			0.048	0.045			0.124 **	0.043		
**ASCOT** Food and drink *Basic*	0.437 **	0.036			0.089 *	0.042			0.128 **	0.040		
**ASCOT** Personal care *Basic*	0.589 **	0.031			0.078	0.046			0.308 **	0.041		
**ASCOT & ASCOT-Carer** Personal safety *Basic*	0.478 **	0.034	0.348 **	0.054	0.177 **	0.042	0.133 *	0.055	0.238 **	0.039	0.094	0.052
**ASCOT & ASCOT-Carer** Control over daily life *Higher Order*	0.587 **	0.030	0.744 **	0.031	0.247 **	0.046	0.205 **	0.065	0.608 **	0.030	0.750 **	0.030
**ASCOT & ASCOT-Carer** Social participation *Higher Order*	0.649 **	0.028	0.682 **	0.036	0.250 **	0.046	0.178 **	0.062	0.691 **	0.027	0.678 **	0.035
**ASCOT & ASCOT-Carer** Occupation *Higher Order*	0.654 **	0.029	0.768 **	0.029	0.299 **	0.048	0.127	0.068	0.727 **	0.026	0.778 **	0.028
**ASCOT** Dignity *Identity*	0.414 **	0.036			0.059	0.041			0.421 **	0.036		
**ASCOT-Carer** Self-care *Basic*			0.629 **	0.039			0.240 **	0.065			0.460 **	0.051
**ASCOT-Carer** Time & space *Identity*			0.803 **	0.027			0.198 **	0.070			0.809 **	0.026
**ASCOT-Carer** Feel supported *Basic*			0.497 **	0.047			0.180 **	0.054			0.260 **	0.052
**Overall QoL**					0.721 **	0.035	0.796 **	0.051				
**EQ-5D Index**					0.469 **	0.035	0.424 **	0.052				
N	737		312		727		311		737		312	
χ ^2^	148.79 **		37.57 **		13.43		33.27 **		28.55 **		14.61	
RMSEA	0.094	**No** ^†^	0.074	**No**	0.036	**Yes**	0.121	**No**	0.038	**Yes**	0.032	**Yes**
95% CI	0.072; 0,107		0.019; 0.089		<.001; 0.055		0.066; 0.171		<.001; 0.046		<.001; 0.045	
90% CI lower	0.080		0.046		<.001		0.083		0.017		<0.001	
90% CI upper	0.108	**No**	0.102	**No**	0.064	**Yes**	0.163	**No**	0.057	**Yes**	0.072	**Yes**
SMSR	0.054	**Yes**	0.042	**Yes**	0.013	**Yes**	0.038	**Yes**	0.022	**Yes**	0.024	**Yes**
95% CI	0.043; 0.063		0.026; 0.051		0.007; 0.016		0.025; 0.052		0.015; 0.026		0.016; 0.029	
CFI	0.888	**No**	0.967	**Yes**	0.986	**Yes**	0.907	**No**	0.980	**Yes**	0.994	**Yes**
95% CI	0.855; 0.928		0.950; 0.997		0.967; 1.018		0.834; 0.973		0.970; 1.000		0.987; 1.000	
TLI	0.784	**No**	0.95`	**Yes**	0.966	**Yes**	0.769	**Yes**	0.969	**Yes**	0.991	**Yes**
95% CI	0.797; 0.898		0.926; 0.996		0.919; 1.018		0.585; 0.932		0.953; 1.002		0.979; 1.015	

*p<0.05, **p<0.01 ¹ **Reflective:** Control, Social, Occupation, Dignity. **Formative:** Accommodation, Food & drink, Personal care, Personal safety. ² **Reflective:** Control, Social, Occupation, Time & space. **Formative:** Self-care, personal safety, Feeling supported. † Goodness of fit criteria met (Yes/No)

**Figure 7.**
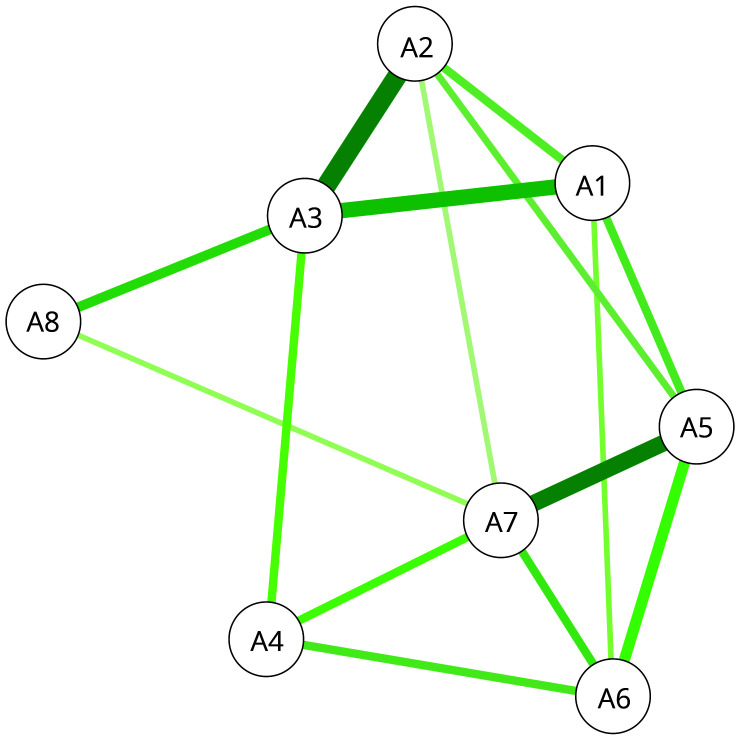
Partial correlation network for ASCOT. **Key:** A1 (Control over daily life) A2 (Social participation) A3 (Occupation) A4 (Safety) A5 (Personal care) A6 (Food and drink) A7 (Accommodation) A8 (Dignity).

**Figure 8.**
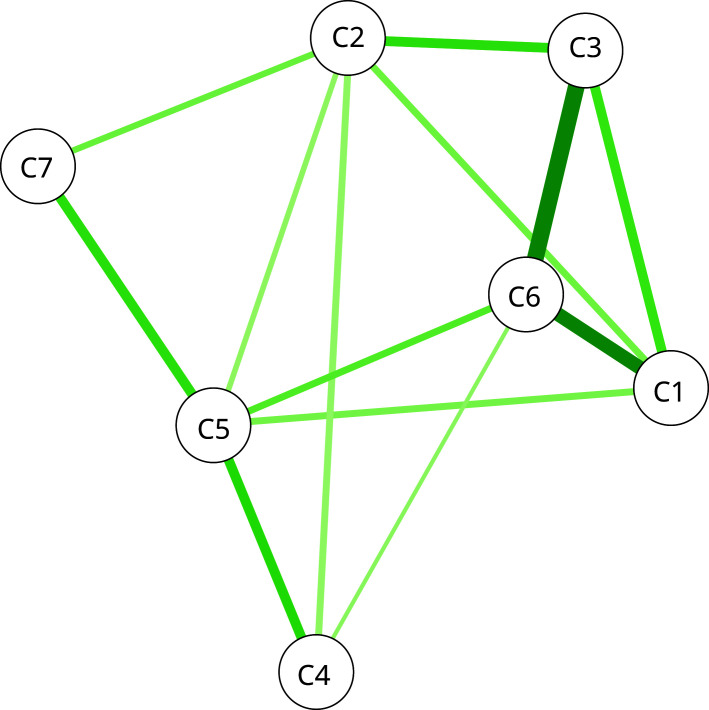
Partial correlation network for ASCOT-Carer. **Key:** C1 (Control over daily life) C2 (Social participation) C3 (Occupation) C4 (Safety) C5 (Self-care) C6 (Time and space to be yourself) C7 (Feeling supported and encouraged).

The network models are shown in Figure 7 and Figure 8. The **nodes** (A1-8, C1-7) represent the items in ASCOT and ASCOT-Carer. The **edges**, shown as lines between nodes, represent empirical correlation between nodes. Thicker lines represent a stronger correlation between items. For both the ASCOT analysis ( Figure 7) and ASCOT-Carer ( Figure 8), there are relevant edges between all nodes. All correlations were positive, shown by green lines.

Degree centrality is an indicator of the relative importance of each node defined by the number of connections connected to each node. This ranged in the **ASCOT analysis** ( Figure 7) from two (Dignity) to five connections (Accommodation). As expected based on the theoretical concept of *higher order domains* and *basic domains*, there are relevant edges between A1 to A3 (higher order) and A5 to A7 (basic), with connection also to A4. The node that relates to the Dignity item has the lowest number of edges, to A3 (Occupation) and A7 (Accommodation), which aligns to the concept that it is distinct from the other nodes since it is the only one that relates to care delivery. The network for **ASCOT-Carer analysis** ( Figure 8) also has a degree centrality from two (Feeling supported) to five (Self-care). However, the structure does not align with the theoretical split between higher order domains (C1 to C3) and basic domains (C4 to C6) as for ASCOT.

The centrality indices for each node are reported in Table 3. These are indicators of the relative importance or influence of each node. Specifically, the indices represent a composite of the number and strength of connections ( *strength*), the relationship with other nodes or its connectivity ( *closeness*) and the importance of a node in relation to the average pathway between other nodes ( *betweenness*). For ASCOT (A1-8), the most influential nodes in ASCOT are Occupation and Personal cleanliness and comfort. Accommodation, Control and Personal safety are also indicated as influential by one of the strength, closeness and betweenness indices, respectively. Since the strength index is least likely to be affected by sample size and is typically the most reliable of the three indices ^29^, we tentatively also highlight Accommodation as an influential node, alongside Occupation and Personal comfort and cleanliness. For ASCOT-Carer (C1-7), there are relevant edges between higher order domains (C1 to C3), but also C6 (Time and Space) to C1 (Control) and C3 (Occupation). The centrality indices indicate the most influential nodes are Time and space, Self-care, Social participation and Occupation.

**Table 3.** Centrality indices.

	Dimension	Higher, basic or identity	Strength	Closeness	Betweenness
	**ASCOT**				
A1	Control over daily life	Higher	0.74	**2.01**	4
A2	Social participation	Higher	0.75	1.87	0
A3	Occupation	Higher	**1.08**	**2.05**	**16**
A4	Personal safety	Basic	0.55	1.93	**6**
A5	Personal comfort & cleanliness	Basic	**0.90**	**1.94**	**6**
A6	Food and drink	Basic	0.70	1.56	0
A7	Accommodation	Basic	**0.94**	1.87	2
A8	Dignity	Identity	0.31	1.39	0
	**ASCOT-Carer**				
C1	Control over daily life	Higher	0.86	2.19	0
C2	Social participation	Higher	0.83	**2.50**	**4**
C3	Occupation	Higher	**0.94**	2.47	**2**
C4	Personal safety	Basic	0.50	1.98	0
C5	Self-care	Basic	**0.91**	**2.56**	**8**
C6	Time and space	Identity	**1.06**	**2.78**	**2**
C7	Feel supported & encouraged	Basic	0.38	1.95	0

**Note.** The three (or four) most central nodes according to each index are reported in bold. Closeness values are multiplied by 100. Values may be compared within, not across, measures.

## Discussion

This study aimed to further understand the nature and internal structure of measures of SCRQoL for adults with social care needs (ASCOT) and their carers (ASCOT-Carer). In the development of the measures and psychometric testing of the original English language version or cultural adaptation and translations, authors have made different assumptions about whether the measure is formative ^1, 2^ or reflective ^15, 16^. In some work, the issue of measurement models has been noted, and an ‘agnostic’ approach taken to analysis that does not commit to either model ^17^. In this study, we have directly compared measurement models for the ASCOT and ASCOT-Carer as *reflective*, *formative* or *mixed*. This provides useful insights to inform the approach for future translation and cross-cultural adaptation of the ASCOT measures.

The findings indicate that the ASCOT and ASCOT-Carer do not empirically fit to the RMM. Based on this empirical finding, especially in combination with the literature on theoretical issues of applying RMMs to PBMs and/or health- or care-related QoL measures, like the ASCOT and ASCOT-Carer, we recommend that the ASCOT measures are not assumed to be reflective. By contrast, the analysis provides tentative empirical evidence of fit of the ASCOT to the formative or mixed measurement model. The former is consistent with the development of the ASCOT, which argued that the attributes should be weakly correlated for its suitability as a PBM ^1^. The fit for the ASCOT-Carer to the formative model is, however, not supported by this analysis. Instead, the best fit is the mixed measurement model. The current guidelines for the development of patient-reported outcome measures (PROMs) suggest that it is acceptable to apply reflective measurement methods ( *i.e*. EFA) to examine structural characteristics of measures based on the mixed measurement model ^8^. This is relevant to cross-cultural adaptation and validation of translations, where EFA is recommended to establish cross-cultural validity ^8^. Therefore, we recommend that future translations of the ASCOT measures may appropriately and usefully apply EFA to explore structural characteristics, especially if the translated version will be applied as a PBM in economic evaluations *and* as a measure of SCRQoL.

In addition to empirically testing the formative, reflective and mixed measurement models, we also applied the network model to explore whether it could add further insights into the nature and structure of ASCOT and ASCOT-Carer. The findings align with the theoretical concept of ASCOT comprising higher order and basic domains that have stronger relationships, as has been found in previous analysis using EFA and correlations for ASCOT ^1^. The former relate to the domains of social participation, occupation and control, which may be conceptualized as aspects of care-related QoL/need beyond basic care-related QoL/needs, like accommodation, food and drink, safety and personal comfort and cleanliness. The analysis presented here for the ASCOT-Carer does not show the same division between basic/higher-order QoL/needs. However, there are key connections between the higher-order domains ( *i.e*. social participation, occupation and control) and time and space to be yourself.

In the analysis presented here, the NM analysis provides useful insights into the nature and internal structure of the measures that add to the insights of established methods, like EFA, CFA or SEM. It does this without specifying the underlying measurement model and in a way that aligns more closely to the complex relationships that are known to exist between aspects of health-related and social care-related QoL ^7^. Specifically, the analysis for ASCOT indicates that Occupation (meaningful and enjoyable activity) and Personal comfort and cleanliness are the most influential aspects of QoL. Accommodation, control over daily life and personal safety are also key nodes. The most influential nodes for the ASCOT-Carer were Time and space, Self-care, Social participation and Occupation. Therefore, we propose that network analysis be used as a complementary approach in development and adaptation of ASCOT measures, alongside psychometric approaches, like EFA, to explore the internal structure and relationship between items.

Furthermore, the NM analysis presented here provides insights that may inform future qualitative research, as well as the application of the measures in care planning and assessment ^7^. This is relevant as there has been interest in applying ASCOT in this way, in England and internationally ^13, 14, 39^. The most influential nodes for each measure and the key relationships between domains may be useful in informing needs assessment and care planning. Specifically, it may guide the conversation to focus on these aspects of QoL for service users (ASCOT) and carers (ASCOT-Carer) respectively, via the target of intervention ( *e.g.* home care), to influence other aspects of QoL for additional benefit. In addition, it may guide research to understand whether and how specific social care interventions are effective. In previous studies, ASCOT measures have been used in qualitative interviews to identify how social care services impact on QoL of adults with care needs and carers ^1, 40, 41^. This research provides insight into which care supports QoL; however, less attention has been given to relationships between QoL domains. The nature of the relationship indicated by an edge in the NM may indicate a direct causal pathway or the common effect of a (latent) variable not included in model ^29^. These associations are indicative of causal relationships that require further investigation, drawing on qualitative evidence, that may then inform the design and evaluation of interventions ^6^.

The study presented here has some limitations. The analysis was conducted on datasets from two studies in England. Further studies to replicate and confirm the findings, both in England and other countries with translated versions of the measure, would add further insight. Study 1 (IIASC) included a diverse sample of users of social care services with a range of needs. However, Study 2 (MOPED) included only a sub-group of carers, *i.e.* of people with dementia, who are known to have higher-level and specific needs compared to other carers. Modelling based on these data may not fully inform the structure of the ASCOT-Carer, when applied for use with carers more broadly, so replication with other groups of carers is important. Furthermore, the specification of the mixed measurement models were limited in the choice of external measures ( *i.e.* EQ-5D and overall QoL rating) by the variables available in the respective study datasets. Although the choice of these measures may be justified by previous studies that show the relationship between SCRQoL and the related constructs of health-related QoL and overall QoL ^1, 12^, it may be that other external measures may be more suitable ( *e.g*. the EQ-VAS or carer-related quality of life or related measures based on the capability approach, like the ICECAP-A ^42^ or ICECAP-O ^43^) and may lead to differences in the coefficients for structural paths and their significance. Indeed, we have not placed much emphasis on interpreting the structural paths, despite being of interest, for this reason.

## Conclusions

The findings of this study indicate that the ASCOT and ASCOT-Carer are not adequately described as RMMs. The ASCOT fits best to either formative or mixed measurement model. ASCOT-Carer fits best to the mixed measurement model. These findings are relevant to the psychometric evaluation of cross-cultural adaptation and validation of translated versions of ASCOT and ASCOT-Carer. We recommend that future translations of the ASCOT measures may usefully and appropriately apply EFA to explore structural characteristics, especially if the translated version will be applied as a PBM in economic evaluations and as a measure of SCRQoL. Further investigation using datasets collected with different populations and in other contexts may usefully guide the approach and provide additional evidence of their internal structure. In addition to EFA, network analysis (based on the network model) may also provide useful insights into the relationships between items.

## List of abbreviations

ASCOT      Adult Social Care Outcomes Toolkit

CFA           Confirmatory factor analysis

CFI            Comparative fit index

EFA           Exploratory factor analysis

FM            Formative measurement model

IIASC       Identifying the impact of adult social care study

LA            Local authority

MIMIC      Multiple indicators multiple causes model

MOPED     Measuring the outcomes of people with dementia and their carers study

NM             Network model

PBM            Preference-based measure

RMM           Reflective measurement model

RMSEA       Root mean square error of approximation

SCRQoL     Social care-related quality of life

SMSR        Standardized root mean square residual

TLI            Tucker-Lewis index

QoL           Quality of life

## Declarations

### Consent

IIASC: Ethical approval for the study was given by the Social Care Research Ethics Committee in England (12/IEC08/0049) with local research governance approval. Written informed consent to participate was obtained from all participants. MOPED: Ethical approval for the study was obtained from the Social Care Research Ethics Committee in England (19/IEC08/0057) with local research governance approval, where carers’ organisations were involved in data collection. Approval to conduct the study in the NHS was granted from the Health Research Authority. Written informed consent to participate was obtained from all participants. 

## Figures and Tables

**Table A.** Exploratory factor analysis.

	ASCOT (Study 1, IIASC)		ASCOT-Carer (Study 2, MOPED)	
	Factor loadings	Uniqueness	Factor loadings	Uniqueness
Accommodation	.63	**.60**		
Food & Drink	.57	**.68**		
Personal comfort & cleanliness	.70	.52		
Personal safety	.54	**.70**	.49	**.76**
Control over daily life	.62	**.61**	.78	.37
Social participation	.68	.54	.73	.46
Occupation	.68	**.53**	.83	.32
Dignity	.44	**.81**		
Self-care			.72	.49
Time & space			.84	.29
Feel supported			.55	**.70**

Items with uniqueness ≥.60 shown in **bold**.

## Data Availability

The datasets generated and/or analysed during the current study are not publicly available due to ethical considerations. Participants did not consent to their full data being shared outside of the research team. Reasonable requests for access to anonymised data will be considered. Please contact s.e.rand@kent.ac.uk. Not applicable.

## Appendix. STATA and R Syntax

*** STATA Syntax (ASCOT)

clear

use "G:\filepath\filename.dta"

*EFA

factortest home food perscare safety control social occ dignity

paran home food perscare safety control social occ dignity, centile(95) iter(5000) quietly graph

polychoric home food perscare safety control social occ dignity

return list

matrix list r(R)

matrix SCRQoL = r(R)

matrix list SCRQoL

factormat SCRQoL, n(770) shape(full) names(home food perscare safety control social occ dignity) pf factor (1)

*CFA

gen scrqol = control + perscare + food + home + safety + social + occ + dignity

*MODEL ONE (reflective)

sem (scrqol -> home food perscare safety control social occ dignity), standardized latent (scrqol)

estat gof, stats(all)

return list

capture program drop fit

program define fit, rclass

sem (scrqol -> home food perscare safety control social occ dignity), standardized latent (scrqol)

estat gof, stats(all)

return scalar cfi=r(cfi)

return scalar tli=r(tli)

return scalar srmr=r(srmr)

return scalar rmsea=r(rmsea)

end

bootstrap cfi=r(cfi) tli=r(tli) srmr=r(srmr) rmsea=r(rmsea), reps(1000) level(95): fit

estat bootstrap, all

*MODEL TWO (formative) MIMIC, with QoL & EQ5D (external)

sem (scrqol -> qol eq5dindex) (home food perscare safety control social occ dignity -> scrqol), standardized latent (scrqol)

estat gof, stats(all)

return list

capture program drop fit

program define fit, rclass

sem (scrqol -> qol eq5dindex) (home food perscare safety control social occ dignity -> scrqol), standardized latent (scrqol)

estat gof, stats(all)

return scalar cfi=r(cfi)

return scalar tli=r(tli)

return scalar srmr=r(srmr)

return scalar rmsea=r(rmsea)

end

bootstrap cfi=r(cfi) tli=r(tli) srmr=r(srmr) rmsea=r(rmsea), reps(1000) level(95): fit

estat bootstrap, all

*MODEL THREE (Mixed) MIMIC

sem (home food perscare safety -> scrqol) (scrqol -> control social occ dignity), standardized latent (scrqol)

estat gof, stats(all)

return list

capture program drop fit

program define fit, rclass

sem (home food perscare safety -> scrqol) (scrqol -> control social occ dignity), standardized latent (scrqol)

estat gof, stats(all)

return scalar cfi=r(cfi)

return scalar tli=r(tli)

return scalar srmr=r(srmr)

return scalar rmsea=r(rmsea)

end

bootstrap cfi=r(cfi) tli=r(tli) srmr=r(srmr) rmsea=r(rmsea), reps(1000) level(95): fit

estat bootstrap, all

*** R Syntax (ASCOT)

#upload .dta file into R

library(foreign)

setwd("G:/filepath")

mydata <- read.dta("ASCOT.dta")

#check variables and view data

names(mydata)

mydata

#pearson correlation matrix with pairwise deletion

cov(mydata, use="pairwise.complete.obs")

#polychoric correlations

library("qgraph")

corMat <- cor_auto(mydata)

#Network analysis

Graph_lasso <- qgraph(corMat, graph = "glasso", layout = "spring", threshold = TRUE, tuning = 0.25,sampleSize = nrow(mydata))

#Calculating centrality indices

centRes <- centrality(Graph_lasso)

# Node strength (degree):

centRes$OutDegree # Or InDegree, it's the same in unweighted networks

# Closeness:

centRes$Closeness

# Betweenness:

centRes$Betweenness

## Appendix. Exploratory factor analysis

### Method

Exploratory factor analysis (EFA) was conducted for ASCOT (Study 1, IIASC) and ASCOT-Carer (Study 2, MOPED). The Kaiser-Meyer-Olkin ( Hutcheson & Sofroniou, 1999; Kaiser, 1974) and Bartlett’s test ( Bartlett, 1954) were applied to assess sampling adequacy and whether the correlations between items were adequate for factor analysis, respectively.

Since ASCOT and ASCOT-Carer are ordinal, polychoric correlations ( Holgado -Tello *et al.*, 2008) using the user-written command, *polychoric* ( Kolenikov, 2016) were applied, and the EFA run on the polychoric correlation matrix. To guide the retention of factors, we applied Horn’s parallel analysis ( Horn, 1965) with more recent Monte Carlo extension to it ( Glorfeld, 1995) with the user-written command, *paran* ( Dinno, 2009; Dinno, 2015). Where the observed Eigenvalue for the factor exceeded the randomly generated Eigenvalue in random correlation matrixes using 95 ^th^ percentile (n=5,000) ( Glorfeld, 1995), the factor was retained. Adjusted Eigenvalues with a value of ≥1 indicated for retention of the factor(s) ( Dinno, 2009). EFA was then run using principal factors with the retained number of factors. The factor structure was then evaluated using the criterion for adequacy (i.e., non-weak loadings) of two or more items with factor loading of ≥.4 ( Hair *et al.*, 1998).

### Results

The exploratory factor analysis is reported in Table A. For both ASCOT and ASCOT-Carer, sampling adequacy (KMO=.82; .87) and correlations between items (Bartlett’s test χ² (28) = 1,166.15, *p* < 0.001; χ² (21) = 733.64, *p* < 0.001) were adequate.

For ASCOT, Horn’s parallel analysis indicated a single factor solution, with the observed higher than random Eigenvalues only for the first factor. The difference was borderline for the second factor, both on visual inspection of the graphical representation ( Horn, 1965) and adjusted Eigenvalues ( Dinno, 2009). (The first component had an adjusted Eigenvalue of 2.91 and the second had an Eigenvalue of .96, which was just under the criterion for retention (adjusted Eigenvalue ≥ 1)). Therefore, we ran the EFA on the polychoric correlation matrix for both one and two factors, separately. The two-factor model applying oblimin rotation (to allow factors to correlate) did not have any factor loadings ≥0.40, so we only report the one-factor solution (see Table A). For the one-factor solution, all factor loadings were ≥0.40. Six of the eight items had very high variance (≥.60), which corresponds to other studies of the structural characteristics of ASCOT, which have observed a single-factor structure with high unique variance ( Netten *et al.*, 2012).

A one-factor solution was also indicated for ASCOT-Carer following Horn’s parallel analysis. Observed Eigenvalues were only higher than random for the first factor (adjusted Eigenvalue = 3.26). The results of the one-factor EFA are reported in Table A. All factor loadings were ≥0.40. Two of the seven items had a very high unique variance (≥.60).
