# Supplementary material for: Exploring the structural characteristics of the Adult Social Care Outcomes Toolkit (ASCOT) and ASCOT-Carer
Source: NIHR Open Res. 2023 Mar 21;2:21. Originally published 2022 Feb 24. [Version 2] doi: 10.3310/nihropenres.13259.2 (PMC10593358; doi:10.3310/nihropenres.13259.2)
Supplement: Peer review history

# Reviewer report: Reviewer response for version 2

**DOI:** 10.3310/nihropenres.14542.r29121

**Published:** 2023-05-02

**Authors:** Herdiantri Sufriyana

**Affiliations:** 1 Graduate Institute of Biomedical Informatics, College of Medical Science and Technology, Taipei Medical University, Taipei, Taiwan; 2 Department of Medical Physiology, Faculty of Medicine, Universitas Nahdlatul Ulama Surabaya, Surabaya, Indonesia

Reviewer revisited only the changes corresponding to the previous comments. Most have been well-addressed with revision. Reviewer let a few comments neither being addressed with revision nor changed with significant amount of revision, considering the correspondence of this peer review is shown to future readers for their own judgment.

 Although there is no revision for major comment #2, authors have explained their standpoints on this matter.

 For minor comment #3, authors also pointed out their previous description for future readers.

 Reviewer appreciate authors’ response for this great work.

Is the work clearly and accurately presented and does it cite the current literature?

Yes

If applicable, is the statistical analysis and its interpretation appropriate?

Partly

Are all the source data underlying the results available to ensure full reproducibility?

Partly

Is the study design appropriate and is the work technically sound?

Yes

Are the conclusions drawn adequately supported by the results?

Partly

Are sufficient details of methods and analysis provided to allow replication by others?

Yes

Reviewer Expertise:

Causal inference from observational data, Statistical and computational machine learning, Pathway (network) analysis.

I confirm that I have read this submission and believe that I have an appropriate level of expertise to confirm that it is of an acceptable scientific standard.

# Reviewer report: Reviewer response for version 2

**DOI:** 10.3310/nihropenres.14542.r29122

**Published:** 2023-03-27

**Authors:** You-Shan Feng

**Affiliations:** 1 Institute for Clinical Epidemiology and Applied Biostatistics, Medical University of Tübingen, Tübingen, Germany

I thank the authors for careful consideration of comments. I have no further concerns at this point and from my point of view, the paper can be approved.

Is the work clearly and accurately presented and does it cite the current literature?

Yes

If applicable, is the statistical analysis and its interpretation appropriate?

Yes

Are all the source data underlying the results available to ensure full reproducibility?

Yes

Is the study design appropriate and is the work technically sound?

Yes

Are the conclusions drawn adequately supported by the results?

Yes

Are sufficient details of methods and analysis provided to allow replication by others?

Yes

Reviewer Expertise:

Clinical Epidemiology, Biostatistics, Psychometrics, Instrument development, Questionnaire scoring, EuroQol Instruments, Self-rated health, Quality of Life Questionnaires

I confirm that I have read this submission and believe that I have an appropriate level of expertise to confirm that it is of an acceptable scientific standard.

# Reviewer report: Reviewer response for version 1

**DOI:** 10.3310/nihropenres.14375.r28794

**Published:** 2022-10-21

**Authors:** Herdiantri Sufriyana

**Affiliations:** 1 Graduate Institute of Biomedical Informatics, College of Medical Science and Technology, Taipei Medical University, Taipei, Taiwan; 2 Department of Medical Physiology, Faculty of Medicine, Universitas Nahdlatul Ulama Surabaya, Surabaya, Indonesia

**General comment**

 This study compared fitting of three methods (i.e., reflective, formative, and mixed) to develop a model for measuring social care-related quality of life (ASCOT) as the construct using several observables. The models were developed for measuring the construct in a care recipient (i.e., ASCOT) or giver (i.e., ASCOT-Carer or ASCOT-C). Subsequently, methodologically validation of these models was conducted by network analysis of the observables. This study is important for future translation and cross-cultural adaptation of the ASCOT measures. Social observables may interact differently across cultures. Structural relationships of the measurement model in the original culture should be described to identify such difference. Nevertheless, several major issues were identified in this study, which may make this study inconclusive yet (please kindly see major comments). Note, reviewer particularly commented on the methodological element of this manuscript, leaving the domain-field element uncommented, due to the limitation of the reviewer’s expertise.

**Major comments**

1. Why were no uncertainty intervals for SMSR and CFI/TLI? To differ if a model indicated a good fit using the criteria from a finding which simply happened by chance (spurious correlation or sampling error), it is important to include the intervals, such as 95% confidence interval. This is possible to be inferred by resampling.
2. How did this study deal with multiple-testing effect? This study only assessed the significant association by p-values, which resulted in many significant findings. Please kindly consider to correct the p-values for multiple-testing effect. Also consider to take effect size into account when identifying the significant association.
3. How was strength, closeness, and betweenness computed? Please briefly explain how to get the numbers. It is also unclear how to deal with these three dimensions to achieve the analytical conclusion.

**Minor comments**

1. Please use terms consistently, e.g.: (1) closeness vs. connectedness which was interchangeably used in the main text and Table 3; (2) ASCOT-Care vs. ASCOT-C; and (3) other terms not limited to these examples.
2. Please kindly consider to classify basic and higher-order domains with sublabels in Tables 2 and 3. Also add sublabels for ASCOT and ASCOT-C in Table 3. It is easier than remembering the alphanumeric codes of the observables.
3. The Introduction and Discussion seem to be written divergently, which makes it difficult to capture the central message. It is still unclear how this methodological comparison plus its network analysis would eventually help for future translation and cross-cultural adaptation of the ASCOT measures. This probably answers the “so-what” of this study.
4. Please kindly consider to share the analytical codes, particularly those by R.

Is the work clearly and accurately presented and does it cite the current literature?

Yes

If applicable, is the statistical analysis and its interpretation appropriate?

Partly

Are all the source data underlying the results available to ensure full reproducibility?

Partly

Is the study design appropriate and is the work technically sound?

Yes

Are the conclusions drawn adequately supported by the results?

Partly

Are sufficient details of methods and analysis provided to allow replication by others?

Yes

Reviewer Expertise:

Causal inference from observational data, Statistical and computational machine learning, Pathway (network) analysis.

I confirm that I have read this submission and believe that I have an appropriate level of expertise to confirm that it is of an acceptable scientific standard, however I have significant reservations, as outlined above.

# Response

**Published:** 2023-03-09

**Authors:** Stacey Rand

**Affiliations:** University of Kent, UK

**Response.** Thank you for your helpful and constructive comments. Our response is below.

**Major comments**

 1. Table 2 now includes the 95% CI estimates for the RMSEA, SMSR and CFI/TFI.

 The text has been edited to clarify that we apply a goodness-of-fit criteria for the RMSEA based on the 90% confidence interval (see Methods, p.9, following Hu & Bentler, 1999), so we also report the 90% CI for the RMSEA.

 In adding the 95% CI estimates, we noticed and corrected two errors in Table 2. (These are shown in track changes for ease of reference). These do not substantively change the results, but we have also highlighted the changes in the report of the results (p.13).

 i. The goodness-of-fit statistics in the MIMIC formative models for ASCOT and ASCOT-Carer were incorrect, due to a formatting error in the previous version. This has led to values showing on the wrong row of the Table, with the omission of standard coefficients/SE for the QoL measures (EQ-5D and QoL item) and the CFI/TLI. These are now correctly presented, along with the 95% CI estimates.

 ii. We realised that there was an inconsistency across the models. The formative models were reported using weighted least squares; the other models using maximum likelihood. For consistency, all analyses are now reported using maximum likelihood, which is what we had originally intended. We have also corrected the description in the Methods section (p.7). The criteria proposed by Hu & Bentler, 1999 – based on ml estimation – are then applied. This does not substantively change the key findings (i.e. that RMM is not a good fit for ASCOT or ASCOT-Carer), even if some of the criteria that were not previously met are now met (i.e. SMSR ≥ .08 for ASCOT and CFI/TLI ≥ .95 for ASCOT-Carer).

 2. The analyses (CFA, MIMIC and network analysis) do not apply any correction for multiple comparisons. The rationale is that the study is designed to understand the structural characteristics of the ASCOT and ASCOT-Carer, against models based on reflective, formative and mixed measurement models. The focus is not on specific factor loadings or standardised coefficients for each item/variable; rather, we are seek to test the fit against the models. This is reflected in the approach in the paper (i.e. a focus on overall model fit, goodness-of-fit statistics).

 3. The centrality indices (strength, closeness and betweenness) were calculated using R (see attached syntax file). These indices are described in the Methods (p.9):

 “Centrality indices were also calculated and reported for node strength, closeness and betweenness. The strength index is a composite measure of both the number and strength of connections to a node. The closeness index represents the relationship between one node and the other nodes through its indirect connections (i.e. its connectedness or connectivity). A high closeness index indicates that the node is affected quickly by changes to the other nodes in the network. The betweenness index indicates the importance of a node in relation to the average pathway between other nodes.”

Reference: Hevey D: Network analysis: A brief overview and tutorial. *Heal Psychol Behav Med.* 2018;6(1):301–28. 34040834 10.1080/21642850.2018.1521283 8114409

 The Results section (p.15) has been edited to improve the clarity of the reported partial correlation networks and their interpretation (Figure 7, 8). The centrality indices are reported in Table 3 and described on pp.15-16.

**Minor comments**

 1. The manuscript has been reviewed and edited for consistency of terminology - specifically, ASCOT-C has been edited to ASCOT-Carer; connectedness to closeness, when referring to the centrality indices.

 2. Tables 2 and 3 have been edited to state which domains relate to ASCOT and/or to ASCOT-Carer, as well as the sublabels of *basic* or *higher order* domains. In Table 3, there are sublabels for each of the domains, alongside the alphanumeric codes.

 3. The introduction (pp.3-5) has been edited to further clarify the link between this study and its application to the psychometric evaluation of translated and cross-cultural adapted PBMs.

 This is also explained in the next paragraph of the Introduction (p.4), as below, as well as the final paragraph of the Introduction (p.5).

 “Inconsistent approaches to psychometric evaluation of the same measure have given rise to discussion of the correct approach to the translation and cross-cultural validity or adaptation of the measures for new contexts or populations 15– 17.“

 This is then restated in the Discussion, paragraph 1 (p.17) and further explored through the Discussion. Some minor edits have been made to the conclusion to, again, clarify how these findings are relevant to translation and cross-cultural adaptation (i.e. they inform our recommendation of the analytic approach to psychometric evaluation of translated and adapted measures).

 4. The STATA and R syntax are provided as an appendix.

# Reviewer report: Reviewer response for version 1

**DOI:** 10.3310/nihropenres.14375.r28513

**Published:** 2022-03-07

**Authors:** You-Shan Feng

**Affiliations:** 1 Institute for Clinical Epidemiology and Applied Biostatistics, Medical University of Tübingen, Tübingen, Germany

The authors present a very interesting paper looking into which model has the best fit with ASCOT data. The study contributes important work to determine how best the items of the ASCOT can be presented and perhaps scored use outside of CUA. The paper is very clearly presented and well written. I also appreciate the detail and presentation of the figures and tables. I have just a few points:

1. I wonder why only 1-factor models were used for the reflective model. Especially as the RMM is found to be the worst fit for the data, it might be prudent to investigate whether an alternative RMM could result in a better fit. Perhaps a 2- or 3-factor model, or a higher order model, would results in worse fit, but this should be discussed in the paper.
2. Why was the preference-based EQ-5D scoring system used instead of a level sum score or using the items as is? Similarly, was the EQ-VAS considered?
3. Given the spread of responses for ASCOT in Study 1, I am very surprised by how low the mean EQ-5D score is: 0.27. The low EQ-5D score should be mentioned in the discussion section as well: is this an expected mean score for this population? How does this and ASCOT responses compare to other patient populations? 
4. A terminology issue: while it is perfectly appropriate to use the term “mixed measurement model” to describe models which include both RMM and FM, the term “mixed model” can be confusing as the terminology is also applied to hierarchal regression methods. I advise authors to consider revising the term “mixed model” for clarity.  

From my point of view, the manuscript is ready for indexing once these few points of concern are addressed.

Is the work clearly and accurately presented and does it cite the current literature?

Yes

If applicable, is the statistical analysis and its interpretation appropriate?

Yes

Are all the source data underlying the results available to ensure full reproducibility?

Yes

Is the study design appropriate and is the work technically sound?

Yes

Are the conclusions drawn adequately supported by the results?

Yes

Are sufficient details of methods and analysis provided to allow replication by others?

Yes

Reviewer Expertise:

Clinical Epidemiology, Biostatistics, Psychometrics, Instrument development, Questionnaire scoring, EuroQol Instruments, Self-rated health, Quality of Life Questionnaires

I confirm that I have read this submission and believe that I have an appropriate level of expertise to confirm that it is of an acceptable scientific standard, however I have significant reservations, as outlined above.

# Response

**Published:** 2023-03-09

**Authors:** Stacey Rand

**Affiliations:** University of Kent, UK

**Response.** Thank you for your helpful and constructive comments. Our response is below.

1. We considered one-factor models based on previous studies using exploratory factor analysis, which have found the ASCOT and ASCOT-Carer to fit to a one-factor model (Netten *et al*, 2012; Rand *et al*, 2019). The manuscript has been edited (p.7.) to explain this. In addition, EFA for the ASCOT and ASCOT-Carer were calculated with the study datasets (IIASC and MOPED, respectively). In both cases, the EFA indicated a one-factor solution.
2. The preference-based EQ-5D scoring system was applied based on current guidance on how to derive an index score from the five EQ-5D-3L/5L item scores for UK studies. Previous studies have found that the ASCOT/-Carer are moderately positively correlated with the EQ-5D-3L, whereas not all items were significantly associated with ASCOT SCRQoL (e.g. the association between ASCOT-Carer and EQ-5D self-care was not significant (Rand et al, 2019)). This informed our decision to focus on the EQ-5D index score. The EQ-VAS was not considered in the analysis. It was only collected in the MOPED study, not the IIASC study. To reflect this, the limitations section has been amended to also refer to the EQ-VAS.
3. The low EQ-5D score reflects the IIASC study aim, design and sample, i.e., it was a study of adults, who use community-based adult social care services (e.g., homecare to help with washing, dressing, eating) due to support needs related to long-term health conditions or disability. ASCOT is a measure of adult social care outcomes designed for completion by adults using adult social care services (e.g. home care). Therefore, studies that apply ASCOT (like IIASC) are likely to have samples with lower EQ-5D scores than general population. The introduction (p.3), methods (p.5) and results (p.10) have been edited to make this clearer.
4. The term ‘mixed model’ has been revised to ‘mixed measurement model’ throughout.
